# A Prospective Controlled Trial of Routine Opt-Out HIV Testing in a Men's Jail

**DOI:** 10.1371/journal.pone.0008056

**Published:** 2009-11-25

**Authors:** Ravi Kavasery, Duncan Smith-Rohrberg Maru, Laurie N. Sylla, David Smith, Frederick L. Altice

**Affiliations:** Section of Infectious Diseases, AIDS Program, Yale University School of Medicine, New Haven, Connecticut, United States of America; University of Sao Paulo, Brazil

## Abstract

**Background:**

Approximately 10 million Americans enter jails annually. The Centers for Disease Control and Prevention now recommends routine opt-out HIV testing in these settings. The logistics for performing routine opt-out HIV testing within jails, however, remain controversial. The objective of this study was to evaluate the optimal time to routinely HIV test newly incarcerated jail detainees using an opt-out strategy.

**Methods:**

This prospective, controlled trial of routine opt-out HIV testing was conducted among 298 newly incarcerated male inmates in an urban men's jail in New Haven, Connecticut. 298 sequential entrants to the men's jail over a three week period in March and April 2008 were assigned to be offered routine opt-out HIV testing at one of three points after incarceration: immediate (same day, n = 103), early (next day, n = 98), or delayed (7 days, n = 97). The primary outcome was the proportion of men in each group consenting to testing.

**Results:**

Routine opt-out HIV testing was significantly higher for the early (53%: AOR = 2.6; 95% CI = 1.5 to 4.7) and immediate (45%: AOR = 2.3; 95% CI = 1.3 to 4.0) testing groups compared to the delayed (33%) testing group. The immediate and early testing groups, however, did not significantly differ (p = 0.67). In multivariate analyses, factors significantly associated with routine opt-out HIV testing were assignment to the ‘early’ testing group (p = 0.0003) and low (bond ≥$5,000, immigration or federal charges or pre-sentencing >30 days) likelihood of early release (p = 0.04). Two subjects received preliminary positive results and one of them was subsequently confirmed HIV seropositive.

**Conclusions:**

In this men's jail where attrition was high, routine opt-out HIV testing was not only feasible, but resulted in the highest rates of HIV testing when performed within 24 hours of incarceration.

**Trial Registration:**

ClinicalTrials.gov NCT00624247

## Introduction

Approximately 10 million Americans enter jails annually [1]. The Centers for Disease Control and Prevention (CDC) recently recommended implementing routine opt-out HIV testing in all healthcare settings, including jails [2]. This presents both a challenge and an opportunity in correctional settings to expand access to HIV services to correctional inmates, a population disproportionately affected by HIV [2], [3], [4]. The CDC has identified several issues that must be addressed when developing model routine opt-out HIV testing strategies in jails [5], including choosing the timing of testing after entering jail.

We have previously reported in this journal the first prospective, controlled trial of routine opt-out HIV testing among female inmates in a jail setting [6]. The objective of this study was to evaluate the optimal time to offer routine opt-out HIV testing in an urban jail setting to newly incarcerated *male* inmates, who represent close to 90% of all jail detainees in the United States.

## Methods

The protocol for this trial and supporting CONSORT checklist are available as supporting information; see Checklist S1 and Protocol S1.

### Ethics Statement

This study was approved by the Institutional Review Board at Yale University School of Medicine and by the Connecticut Department of Correction Research Committee.

### Design Overview

The study design, eligibility criteria, subject allocation, study procedures, definitions, outcome measures and analytic approach have been previously described for a similar trial in a women's jail [6]. For this trial, all 298 consecutive, newly incarcerated male inmates from March 25, 2008 to April 16, 2008 were offered routine opt-out HIV testing after being sequentially assigned to one of three study arms upon admission to the facility: 1) ‘immediate’ (during an initial medical screen the night of admission); 2) ‘early’ (during a physical exam the following evening); or 3) ‘delayed’ (7 days after arrival to the facility).

### Setting and Participants

This prospective, controlled trial was conducted at the New Haven Community Correctional Center (NHCCC) in New Haven, Connecticut, an urban men's jail that houses primarily unsentenced detainees as well as those serving sentences ≤1 year. The facility's average daily census is 919 individuals. Similar to other jails, a brief, standardized medical and psychiatric assessment is routinely conducted on all inmates, including medical, sexual, and drug-use histories immediately upon arrival. Voluntary HIV testing is available by medical referral or by self-request and often involves being placed on a waiting list. Current policy in Connecticut requires that in the absence of an emergent clinical indication, inmates must be beyond the three month “window period” from their last HIV risk behavior to receive an HIV antibody test. Newly confirmed HIV positive test results are reported to the Connecticut Department of Public Health as part of the state's mandatory reporting system.

## Results

The baseline characteristics of the study population appear in Table 1. The 298 newly incarcerated men were sequentially assigned to the following testing groups: ‘immediate’ (N = 103, the night of admission), ‘early’ (N = 98, the following evening), and ‘delayed’ (N = 97, 7 days later). The three study groups did not differ significantly with respect to any of the social and demographic characteristics assessed.

**Table 1:** Baseline Characteristics of the Study Population (n = 298).

Characteristics	Subcategory	Value (%)
Age (mean years; SD)		35 (11)
Ethnicity	Hispanic	56 (19)
	Black	104 (35)
	White/Other	138 (46)
High School Graduate	Yes	193 (65)
	No	105 (35)
Length of Current Incarceration (median days; IQR)		28 (4–36)
High Likelihood of Early Release*	Yes	122 (41)
	No	176 (59)
Drug- or Prostitution-Related Offense	Yes	46 (15)
	No	252 (85)
Previous History of Incarceration	Yes	244 (82)
	No	54 (18)
Has Medical Insurance	Yes	276 (93)
	No	22 (7)

*High: any charges directly related to prostitution or drugs.

Low: bond value ≥$5000, bond sentencing >30 days, immigration or federal charges, or no bond.

**Figure 1:**
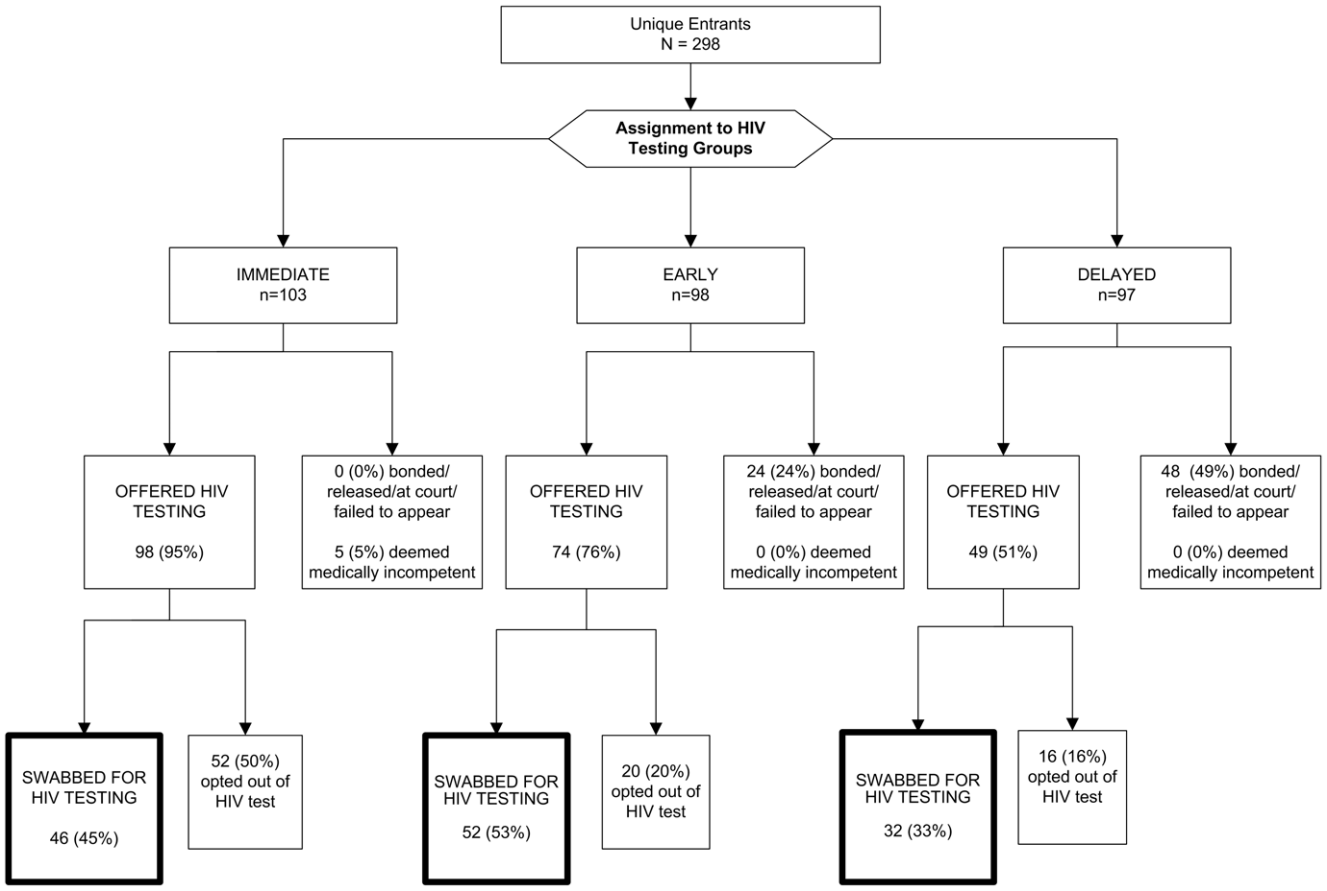
Disposition of Inmates Approached for HIV Testing.

**Figure 2:**
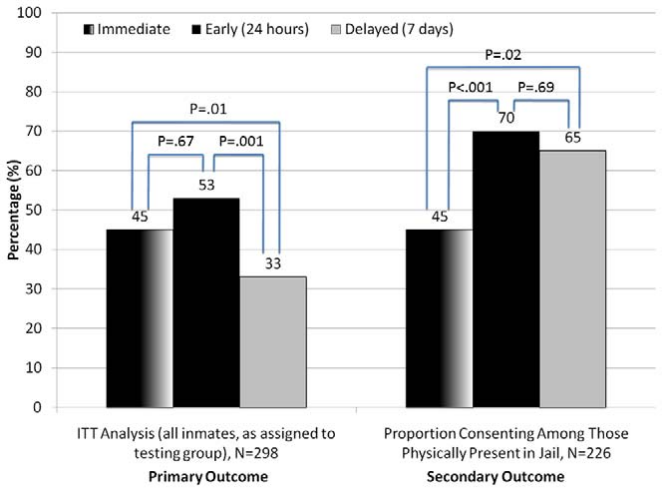
Rapid HIV Testing Swab Results by Assigned Testing Group.

The disposition of individuals approached for routine opt-out HIV testing in this trial is illustrated in Figure 1. Overall, 130 (44%) of 298 inmates assigned to testing groups provided verbal consent to be swabbed for routine opt-out HIV testing. Among those assigned to early testing, 52 (53%) accepted HIV testing versus 46 (45%) in the immediate and 32 (33%) for 7 days post-entry groups (Figure 2). Compared to the delayed testing group, the early (OR = 2.6; 95% CI = 1.5 to 4.7; p = 0.001) and immediate (OR = 2.3; 95% CI = 1.3 to 4.0; p = 0.01) testing groups were significantly more likely to be swabbed for HIV testing. The immediate and early testing groups did not differ with regard to the primary outcome (p = 0.67).

There were differences between these two groups, however, in rates of acceptance among those actually physically available and medically competent to be approached for testing. Of the 226 subjects that were physically present in the jail at each of the three time points, acceptability was highest for the early testing group (N = 52/74, 70%), compared to 45% (N = 46/103) and 65% (N = 32/49) in the immediate and delayed testing groups, respectively (see Figure 2).

**Figure 3:**
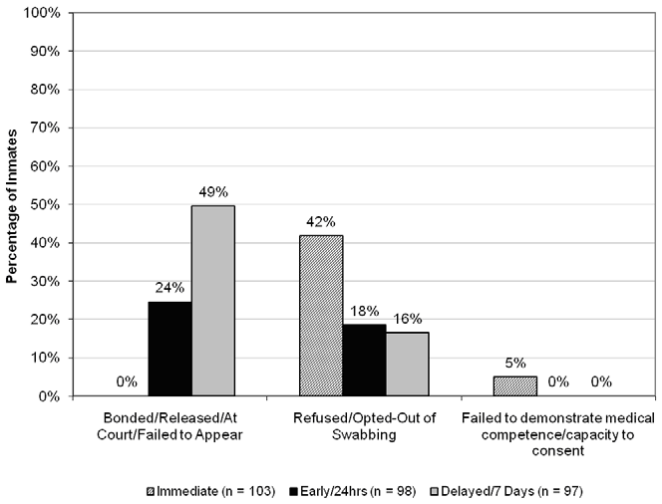
Reasons Inmates were not Swabbed for HIV Testing.

Stratified by testing group assignment, the reasons that inmates were not swabbed are depicted in Figure 3. In the immediate group, 5 (10% of those not swabbed in that group) were medically incompetent or did not have the capacity to consent, compared with none in the ‘early’ and in the ‘delayed’ testing groups. In the ‘delayed’ testing group, 48 (75% of those not tested) were no longer available for testing compared with none in the ‘immediate’ and 24 (57% of those not tested) in the ‘early’ groups. Among the 77 competent subjects who declined testing, 18 (23%) stated they were not interested in general, 15 (19%) did not perceive themselves to be at risk, 12 (16%) self-reported they were already HIV-infected (confirmed by medical record review), and 11 (14%) stated they were recently tested.

**Figure 4:**
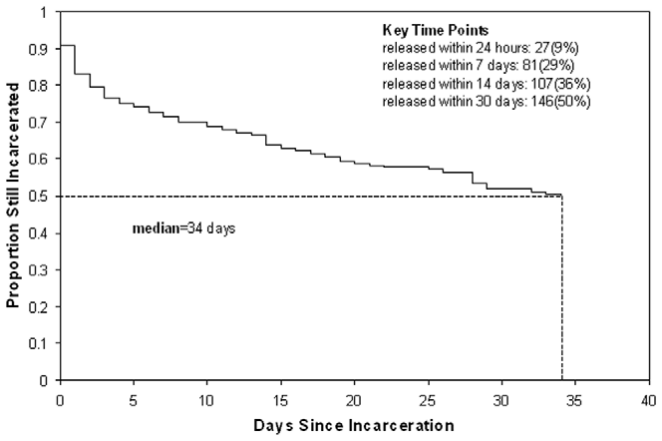
Time to Release Following Incarceration.

Figure 4 demonstrates the time to release from the facility. The median duration of incarceration at the facility was 34 days. Among the 298 subjects approached, 51 (17%) were released within the first 24 hours following admission, 81 (29%) were no longer incarcerated after 7 days, 107 (36%) after 14 days, and 142 (48%) were already released at 30 days. Individuals released within the first 24 hours following admission were less likely to have been incarcerated previously: 33 (65%) versus 211 (85%) (p = 0.0005); the two groups did not vary on any other characteristics.

Bivariate and multivariate analyses were conducted to determine predictors associated with being swabbed for routine opt-out HIV testing (Table 2). In the bivariate analysis, assignment to the ‘immediate’ or ‘early’ testing groups was associated with being swabbed for HIV testing. In the multivariate analysis, however, only assignment to the ‘early’ testing group (p = 0.0003) and low likelihood of release (p = 0.04) were significantly associated with being swabbed for HIV testing.

**Table 2:** Bivariable and Multivariable Predictors of Receipt of Swab.

	*Uptake Rates, n (%)*	*Bivariable OR (95% CI)*	*Bivariable p-value*	*Multivariable OR (95% CI)*	*Multivariable p-value*
Assigned day 0*	55 (53)	2.3 (1.26 to 4)	0.01	2.4 (1.4 to 4.3)	0.00
Assigned day 1*	56 (57)	2.6 (1.5 to 4.7)	0.0013	**3.0 (1.7 to 5.6)**	**0.0003**
Assigned day 7*	33 (34)	–Referrent–	–	–Referrent–	–
Low Likelihood of Release	52 (43)	0.7 (0.5 to 1.1)	0.10	**0.1 (0.1 to 0.7)**	**0.04**
High Likelihood of Release	92 (52)	–Referrent–	–	–Referrent–	–
High School Graduate	88 (46)	0.74 (0.5 to 1.2)	0.20	0.8 (0.5 to 1.2)	0.21
Not High School Graduate	56 (53)	–Referrent–	–	–Referrent–	–
Has Medical Insurance	131 (47)	1.6 (0.7 to 3.9)	0.30	2 (0.8 to 5.1)	0.15
No Medical Insurance	13 (59)	–Referrent–	–	–Referrent–	–
Age (years) at Entry**	–	0.9 (0.72 to 1.12)	0.32	-Out of Model-	–
Low HIV-Risk Offense	18 (39)	–Referrent–	–	-Out of Model-	–
High HIV-Risk Offense	126 (50)	0.7 (0.4 to 1.3)	0.18	-Out of Model-	–
White/Other	49 (47)	–Referrent–	–	-Out of Model-	–
Black	29 (52)	1 (0.6 to 1.5)	0.73	-Out of Model-	–
Hispanic	66 (48)	1.2 (0.7 to 2.2)	0.58	-Out of Model-	–
No Previous Incarceration	23 (43)	–Referrent–	–	-Out of Model-	–
Previous Incarceration	121 (50)	1.4 (0.8 to 2.5)	0.35	-Out of Model-	–

*OR Comparing day 1 or day 7, respectively to day 0.

**The calculated OR represents the added likelihood conferred by every 10 years of age.

Of the 144 individuals swabbed, 130 (90%) provided written consent to complete the entire study. Of these, 128 (98.5%) were HIV-negative and 2 (1.5%) had a preliminary positive test result; one was a false-positive and the other was confirmed using Western Blot testing. The one confirmed negative test occurred in the “immediate testing group”. Both individuals who tested preliminary positive were incarcerated at seven days and both received their confirmatory test results. Based on the 12 confirmed individuals known to be HIV-infected and the one newly diagnosed subject in this study, the minimum HIV prevalence for this facility is 13/298 (4.4%).

Among the 130 HIV-tested subjects who underwent standardized screening, 15 (12%) exhibited moderate or severe opioid withdrawal symptoms: 6 (13%) from ‘immediate’, 4 (8%) from ‘early’ group, and 5 (16%) from the ‘delayed’ testing group. Only 3 (2%) individuals were deemed to have increased risk for alcohol withdrawal symptoms: 1 (2%) from ‘immediate’, 2 (4%) from ‘early’, and none from the ‘delayed’ testing group. In addition, 17 (13%) of the 130 tested subjects had evidence of serious mental illness using the K6 psychological distress scale score: 5 (11%) from ‘immediate’, 9 (17%) from ‘early’, and 3 (9%) from the ‘delayed’ testing group.

## Discussion

This study reports the first prospective controlled trial of routine HIV testing in a men's jail. Our results have two major public health implications. First, routine opt-out HIV testing in jails is feasible, whether provided immediately upon intake or a day or week later. The operational details of our program should provide guidance to jails implementing routine opt-out HIV testing. The characteristics of the available jail population vary over time such that delays in testing result both in reduced likelihood of testing but also missing the important opportunity to HIV test those who have never interfaced with the correctional environment. Second, and perhaps most importantly, the primary outcome from this trial demonstrated that offering routine opt-out HIV testing to male inmates in this urban jail within the first 24 hours of admission resulted in the highest likelihood of being HIV tested (53%). This suggests that routine opt-out HIV testing in jails should be offered as early in the intake process as possible. To balance the competing factors of risk of release with inmate willingness to accept testing, it may be beneficial to offer routine opt-out HIV testing at intake and again at a subsequent medical appointment within 24 hours if the inmate is not tested the night of intake.

We previously reported in this journal the first prospective, controlled trial of routine opt-out HIV testing among female inmates using a similar study design [6]. In that trial, the proportion of subjects consenting to be swabbed and tested for HIV was significantly highest 24 hours after admission compared to testing immediately upon intake (OR = 2.3; 95% CI = 1.3–4.0; p = 0.005) or 7 days post-entry (OR = 2.7; 95% CI = 1.5–4.7; p = 0.0007). The proportion of individuals choosing to opt out of testing the night of admission was high in both the women's and men's studies (32% and 50%, respectively),. Similarly, inmates in both trials were considerably more willing to accept testing when offered the day after entry. In this trial, among inmates physically present in the jail at the time of testing, 55% of those approached in the immediate group opted out, versus 30% in the early group. The attrition rate due to inmates quickly bonding out resulted in the equalization of swabbing rates between these two groups.

In contrast to the women's jail, however, this trial among male inmates was conducted at an urban facility with more daily admissions and a higher rate of release within the first twenty-four hours. These dynamics of increased attrition in the men's jail may account for the lost benefit of waiting until the day following entry to maximize uptake of HIV testing.

Prior to this study and our trial of routine opt-out HIV testing in a women's jail, published works regarding routine opt-out HIV testing in jail settings have been limited [7], [8]. One observational study conducted recently in a Rhode Island jail demonstrated markedly higher rates of acceptability of testing compared to that found in our study. The likely explanation for this difference is that mandatory HIV testing of prisoners has been in place in that state for nearly 20 years. As such, nearly all (88%) subjects had previously been tested within that setting and the authors themselves suggest that HIV testing was no longer considered as an emotional or “charged” issue. It can therefore be expected that acceptance of routine opt-out HIV testing will increase with time as the stigma and unfamiliarity with testing decreases among correctional staff and inmates. Additionally, a 4-site, CDC funded study demonstrated that rapid, voluntary HIV testing is feasible and identified many new people living with HIV. However, inmates often were tested days to weeks after incarceration, thereby potentially missing a large number of high risk individuals who were released prior to testing [9]. New initiatives examining HIV testing strategies in jails are now underway [10].

In addition, a key factor contributing to the higher rate of routine opt-out HIV testing within the first 24 hours of admission was that the testing procedures were linked to a routine clinical activity (intake or physical exam) with clinical personnel. This policy, of linking routine opt-out HIV testing with routine clinical activities, makes logistical sense and should be considered when implementing testing in the future. It also helps to demonstrate to inmates that HIV testing is simply a component of comprehensive primary healthcare. Future observational and controlled studies should assess which staff members should perform testing and delivery of both positive and negative HIV test results during the chaotic post-entry period. Our study did not assess this fully; we utilized jail staff for intake, testing, and follow-up of positive HIV test results, while all our own research staff provided negative results.

As is typical of many urban jails in the United States, this facility houses a population with rapid turnover. Nearly a fifth of new admissions were released by the next day, with 29% no longer remaining in the facility within a week. This raises significant questions about the current policy in Connecticut of requiring HIV testing only on those inmates with at least 90 days since their last HIV risk behavior. Continuing such a policy would result in nearly three quarters of jail detainees being ineligible for HIV testing because they would already be released. Delivery of test results, particularly for individuals who have blood drawn for confirmatory testing, will prove difficult among this transient population. In this study, only one individual was released prior to receiving his confirmatory negative test result, and both individuals who tested preliminarily positive were incarcerated at 7 days to receive their final test results. If routine opt-out HIV testing is to be broadly implemented in our nation's jails, however, delivery of test results will remain an important issue and requires further resolution. Logistical issues of providing results in the jail will be incumbent on correctional authorities to resolve, yet public health infrastructure must be maintained to address case finding and delivery of results to those who leave before HIV testing results are available.

An additional important finding was that those having a low likelihood for release were more likely to consent to testing, regardless of group assignment. This suggests that, in jail systems with high volumes that preclude testing of all inmates at entry, triage systems could be useful in focusing initial testing efforts on those inmates for whom early release is more likely. While this study did diagnose one new individual with HIV, it likely missed many other high risk individuals who left the facility before being offered testing as part of their assigned testing group.

There are several important limitations of the present study. Owing to logistical difficulties, we could not undertake a true randomized trial. This makes it possible that confounders, such as cohort effects from particular peer leaders' influence on testing uptake, biased our results (internal validity). Our large sample size and final effect size suggests, however, that the differences detected here were real. Additionally, since our trial was conducted at only one men's jail, its external validity will itself have to be assessed by studies from other sites.

In conclusion, our study confirms that routine opt-out HIV testing in a jail setting is feasible and that early testing will likely result in the largest number of individuals being tested. Early testing also results in testing a larger proportion of those who have never been within the correctional system before and have previously received an HIV test. Such programs, if implemented properly, will result in identifying individuals with HIV who do not know they are infected and increase their likelihood of reducing their HIV risk behaviors and increasing their access to HIV treatment and prevention services.

## Supporting Information

Checklist S1 CONSORT Checklist
(0.06 MB DOC)

Protocol S1 Trial Protocol
(0.43 MB DOC)
